# Regional Variation in Aortic AT1b Receptor mRNA Abundance Is Associated with Contractility but Unrelated to Atherosclerosis and Aortic Aneurysms

**DOI:** 10.1371/journal.pone.0048462

**Published:** 2012-10-31

**Authors:** Aruna Poduri, A. Phillip Owens, Deborah A. Howatt, Jessica J. Moorleghen, Anju Balakrishnan, Lisa A. Cassis, Alan Daugherty

**Affiliations:** 1 Saha Cardiovascular Research Center, University of Kentucky, Lexington, Kentucky, United States of America; 2 Department of Molecular and Biomedical Pharmacology, University of Kentucky, Lexington, Kentucky, United States of America; 3 Graduate Center for Nutritional Sciences, University of Kentucky, Lexington, Kentucky, United States of America; University of Freiburg, Germany

## Abstract

**Background:**

Angiotensin II (AngII), the main bioactive peptide of the renin angiotensin system, exerts most of its biological actions through stimulation of AngII type 1 (AT1) receptors. This receptor is expressed as 2 structurally similar subtypes in rodents, termed AT1a and AT1b. Although AT1a receptors have been studied comprehensively, roles of AT1b receptors in the aorta have not been defined.

**Methodology/Results:**

We initially compared the regional distribution of AT1b receptor mRNA with AT1a receptor mRNA in the aorta. mRNA abundance of both subtypes increased from the proximal to the distal aorta, with the greatest abundance in the infra-renal region. Corresponding to the high mRNA abundance for both receptors, only aortic rings from the infra-renal aorta contracted in response to AngII stimulation. Despite the presence of both receptor transcripts, deletion of AT1b receptors, but not AT1a receptors, diminished AngII-induced contractility. To determine whether absence of AT1b receptors influenced aortic pathologies, we bred AT1b receptor deficient mice into an LDL receptor deficient background. Mice were fed a diet enriched in saturated fat and infused with AngII (1,000 ng/kg/min). Parameters that could influence development of aortic pathologies, including systolic blood pressure and plasma cholesterol concentrations, were not impacted by AT1b receptor deficiency. Absence of AT1b receptors also had no effect on size of aortic atherosclerotic lesions and aortic aneurysms in both the ascending and abdominal regions.

**Conclusions/Significance:**

Regional abundance of AT1b receptor mRNA coincided with AngII-induced regional contractility, but it was not associated with AngII-induced aortic pathologies.

## Introduction

Angiotensin II (AngII) is the major effector of the renin angiotensin system that exerts its actions predominantly through stimulation of AT1 receptors [1]–[3]. In rodents, this receptor undergoes chromosomal duplication and is expressed as two subtypes, named AngII type 1a (AT1a) and type 1b (AT1b) receptors [4]. These two receptor subtypes are present on different chromosomes, 13 and 3 for AT1a and AT1b in mice, respectively. While AT1a receptors have ubiquitous tissue expression, AT1b receptor expression is more restricted to tissues such as aorta, resistance arteries, adrenal glands, pituitary, and hypothalamus [2], [5]–[8]. The aorta is one of the few tissues that express both subtypes, presumably in medial smooth muscle cells (SMCs) [2].

AT1 receptor subtypes in rodents are highly homologous, both containing 359 amino acids that have 94% similarity [9], [10]. AngII stimulation of both subtypes are inhibited indiscriminately by the sartan class of pharmacological inhibitors [9], [10]. However, the consequences of AngII stimulation of the two subtypes could vary in the same cell type as several of the amino acid differences between the two subtypes are clustered in the intracellular domain of the third loop and the cytoplasmic tail. Despite not being characterized yet, amino acid substitutions in these structural positions have the potential to impart differences on multiple signaling pathways that are invoked by AngII stimulation of AT1 receptors [1].

AngII provokes region-specific effects on the aorta. Ex vivo, this heterogeneity is reflected by differences in AngII promoting aortic contractility with only the abdominal region being responsive [2], [3], [11]. Recently, it has been demonstrated that aortic contraction induced by AngII is restricted to the infra-renal region of the abdominal aorta [3]. However, it is unclear whether this region-specific effect is related to the relative abundance of the receptor subtypes along the length of the aorta.

AngII infusion augments atherosclerosis in hypercholesterolemic mice [12], [13]. AngII infusion also promotes aortic aneurysms in both the ascending and supra-renal regions, which represent two distinct pathologies [12], [14]. Whole body AT1a receptor deficiency attenuates atherosclerosis and aortic aneurysms in mice infused with AngII [3], [15]. In contrast to AT1a receptors, AT1b receptors regulate calcium signaling in vascular smooth muscle cells of the aorta and blood pressure in response to AngII stimulation [16], [17], indicating a potentially important role of AT1b receptors in vascular diseases. Although AT1b receptors are also an AngII binding receptor that is abundant in the aorta, it has not been determined whether this receptor subtype also influences atherosclerosis and aortic aneurysms in mice infused with AngII.

AT1b receptor mRNA has been detected in the aorta [2]. However, no study has reported whether there are regional differences of AT1b receptor mRNA in the aorta. In the present study, we initially quantified the mRNA abundance of AT1a and AT1b receptors in distinct aortic regions. Both subtypes were primarily expressed in the infra-renal aortic region. Despite the presence of transcripts for both receptors in this region, deletion of AT1b receptors, but not deletion of AT1a receptors, inhibited AngII-induced contractility. In contrast to the major role of AT1a receptors, deletion of the AT1b receptor subtype had no effect on AngII-induced atherosclerosis and aortic aneurysms.

## Materials and Methods

### Ethics Statement

All mouse studies were performed with approval of the University of Kentucky Institutional Animal Care and Use Committee (IACUC number: 2006-0009). Pump implantation was conducted using isoflurane inhalation anesthesia, and termination was performed with overdose of ketamine/xylazine. All efforts were taken to minimize suffering to mice in accordance with the University of Kentucky IACUC and the Care and Use of Laboratory Animals per the National Institutes of Health guidelines.

### Mice

Male low density lipoprotein (LDL) receptor −/− (B6.129S7-ldlr^tm1her^; Stock# 002207) and AT1a receptor −/− (B6.129P2-Agtr1a^tm1Unc^; Stock# 002682) mice were obtained from The Jackson Laboratory (Bar Harbor, ME, USA). AT1b receptor −/− mice in a C57BL/6 background were generated at Duke University (Durham, NC, USA) and were a generous gift from Dr. Thomas Coffman [18]. All mice were backcrossed 10 times into a C57BL/6 background. Male AT1b receptor −/− mice were mated to female LDL receptor −/− mice to generate heterogeneous progeny. The F2 progeny were screened for developing breeding pairs of AT1b receptor +/+ or −/−×LDL receptor −/− mice. AT1b receptor +/+ and −/− mice in the LDL receptor −/− background were used for the present study. Mice were maintained in a barrier facility and fed a standard laboratory diet unless specified otherwise.

### Real-time PCR

RNA was isolated from aortic tissues of C57BL/6 mice by RNeasy fibrous tissue kit (Cat# 74704, Qiagen, Valenica, CA, USA). RNA was reversely transcribed using an iScript cDNA synthesis kit (Cat# 170–8891, Bio-Rad, Hercules, CA, USA). Real time PCR was performed using a CFX96 cycler (Bio-Rad). Taqman probes (ABI, Carlsbad, CA, USA) specific for AT1a and AT1b receptors, respectively, were used to measure mRNA abundance. mRNA abundance in the selected aortic regions was calculated with 18S rRNA normalization using the ΔΔC_t_ method. Samples containing either no template or no RT reactions were used as negative controls.

### Genotyping

DNA was isolated from tail tissues using a DNeasy blood and tissue kit (Cat# AS1120, Promega, Madison, WI, USA). Mouse genotypes were determined by polymerase chain reaction (PCR). AT1b receptor genotyping was determined using the following primers: 5′-GCATCATCTTTGTGGTGGG and 5′-ATGAGCACATCCAGAAAA C. The PCR was performed with 1 step of 94°C for 5 min; 35 cycles of 94°C for 1 min, 55°C for 1 min, and 72°C for 2 min; and 1 step of 72°C for 5 min. Wild type and disrupted alleles generated amplicon sizes of 550 bp and 1,600 bp, respectively. AT1a receptor and LDL receptor genotypes were verified by PCR as described previously [19].

### Aortic Contractility

Mice were anesthetized with ketamine (100 mg/kg) and xylazine (10 mg/kg) as described previously [20]. Aortas from C57BL/6, AT1a receptor −/−, and AT1b receptor −/− mice were perfused with saline via the left ventricle and then removed, and adventitia were cleaned off completely without damages of the media portion. Measurement of contractile activity was performed using infra-renal aortic rings as described previously [3]. Infra-renal segments were mounted by passing two tungsten wires through the arterial lumen and arterial segments were immersed in Krebs Henseleit solution. Tension (1 gram) was maintained continuously and recorded with a Micro-Med Tissue Analyzer (TFA-410, Louisville, KY, USA). After a 30 min equilibration, tissues were incubated with potassium chloride (KCl; 80 mM, Sigma, St Louis, MO, USA), 5-hydroxytryptamine (5-HT; 1 µM, Sigma), or AngII (1 µM, Sigma) for 30 min between the agonists.

### Diet and AngII Infusions

To induce hypercholesterolemia, mice were fed a diet supplemented with saturated fat (milk fat 21% wt/wt) and cholesterol (0.2% wt/wt; Diet# TD.88137, Harlan Teklad, Madison, WI, USA) ad libitum starting 1 week prior to pump implantation, and continuing for the 28 days of saline or AngII infusion. After 1 week of feeding, AngII (1,000 ng/kg/min; Cat# H-1705, Bachem, Torrance, CA, USA) was infused subcutaneously into 8–10 week old male mice subcutaneously via Alzet osmotic minipumps (Model 2004, Durect Corporation, Cupertino, CA, USA) for 28 days as described previously [12], [21].

### Systolic Blood Pressure Measurements

Systolic blood pressure (SBP) was measured during the last week for 5 consecutive days of AngII infusion using a non-invasive tail cuff system (Coda 8, Kent Scientific Corporation, Torrington, CT, USA) on conscious mice as described previously [22].

### Plasma Component Measurements

Blood was drawn by right ventricular puncture and collected in EDTA (1.8 mg/ml) coated vials at termination. Plasma cholesterol concentrations were quantified with a commercially available enzymatic kit (Cat# Cholesterol E 439–17501, Wako Chemical USA, Richmond, VA, USA) as described previously [23]. Plasma renin concentrations were measured by radioimmunoassay using a commercially available kit (Cat# 1553, DiaSorin Inc., Stillwater, MN, USA) as described previously [20].

### Atherosclerosis and Aortic Aneurysm Quantification

After exsanguination, saline was perfused through the left ventricle of the heart. Aortas were removed and kept in neutrally buffered formalin (10% w/v) overnight before adventitia were cleaned off completely. Thoracic aorta was cut open and pinned. Aortic arch region included the ascending aorta, aortic arch, and 3 mm distal from the aortic orifice of the left subclavian artery. The remaining length of the thoracic aorta till the last intercostal branches was defined as the descending thoracic aorta. Atherosclerosis was quantified on the intima of both the arch and descending thoracic regions as described previously [24]. Oil Red O staining of aortas was performed as described previously [24], [25].

Luminal diameters of abdominal aortas were measured in vivo with a high frequency ultrasound (Vevo 660, VisualSonics, Toronto, Canada) on day 0 (the day before osmotic minipump implantation) and day 28 during AngII infusion as described previously [26]. This system acquired images that were used to measure maximal lumen dilation. After termination, aortas were cleaned off adventitia and pinned, and maximal external supra-renal diameters were measured using Image-Pro Software 7.0 (Media Cybernetics Inc., Bethesda, MD, USA) as described previously [27].

After measurements of ex vivo diameter, supra-renal aortas were embedded in optimal cutting temperature compound and serially sectioned in a cryostat. Tissues were sectioned in a serial set of 8 slides that contained 9 sections (thickness: 10 µm/section) of each slide. To characterize AAAs, Movat’s pentachrome staining was performed as described in our previous studies [28].

### Statistical Analyses

SigmaPlot 12.0 (SYSTAT Software Inc., San Jose, CA, USA) was used for statistical analyses. Data were represented as mean ± standard error of mean (SEM). Student’s *t* test or Mann-Whitney Rank Sum test was performed as appropriate for two-group comparisons, and one way ANOVA was performed for multiple-group (more than 2 groups) comparisons. Two way ANOVA with Holm-Sidak post hoc analysis was performed for multiple-group and multiple-manipulation analysis. Two way repeated measures ANOVA was conducted to compare aortic diameters measured on day 0 and day 28 using ultrasonography. A P<0.05 was considered statistically significant.

## Results

### mRNA Abundance of Both AT1 Receptor Subtypes was Increased in Infra-renal Aortas

**Figure 1:**
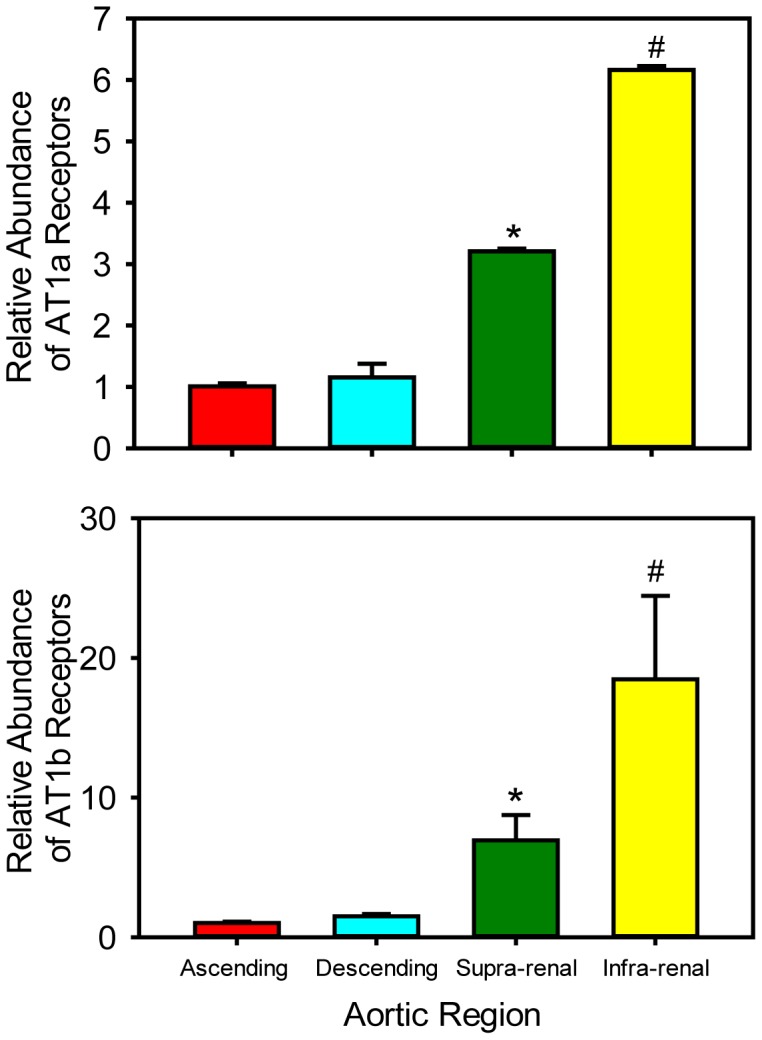
Region-specific differences in aortic mRNA abundance of AT1a and AT1b receptors. mRNA abundance of (**A**) AT1a receptors (N = 4) and (**B**) AT1b receptors (N = 4) in aortic regions of C57BL6/J mice. * denotes P<0.05 in supra-renal versus ascending and descending aortic regions (one way ANOVA with Holm-Sidak post hoc test). # denotes P<0.001 in the infra-renal region versus all the other aortic regions (one way ANOVA with Holm-Sidak post hoc test).

We first measured mRNA abundance of both receptor subtypes throughout the aorta by real-time PCR. mRNA abundance of receptor subtypes was increased significantly in the infra-renal aorta compared to other selected regions of aortas (Figure 1A and 1B; P<0.001). Compared to the infra-renal region, mRNA abundance of both receptor subtypes was much lower in supra-renal aortas, and very low in both ascending and descending aortic regions (Figure 1A and 1B). In agreement with previous studies [2], [5], [8], we observed higher relative abundance of AT1a receptor mRNA in liver and kidney, while AT1b receptor mRNA was either not detected or negligible in these tissues (data not shown).

### AT1b Receptor Deficiency Abolished AngII-induced Infra-renal Contractile Response

**Figure 2:**
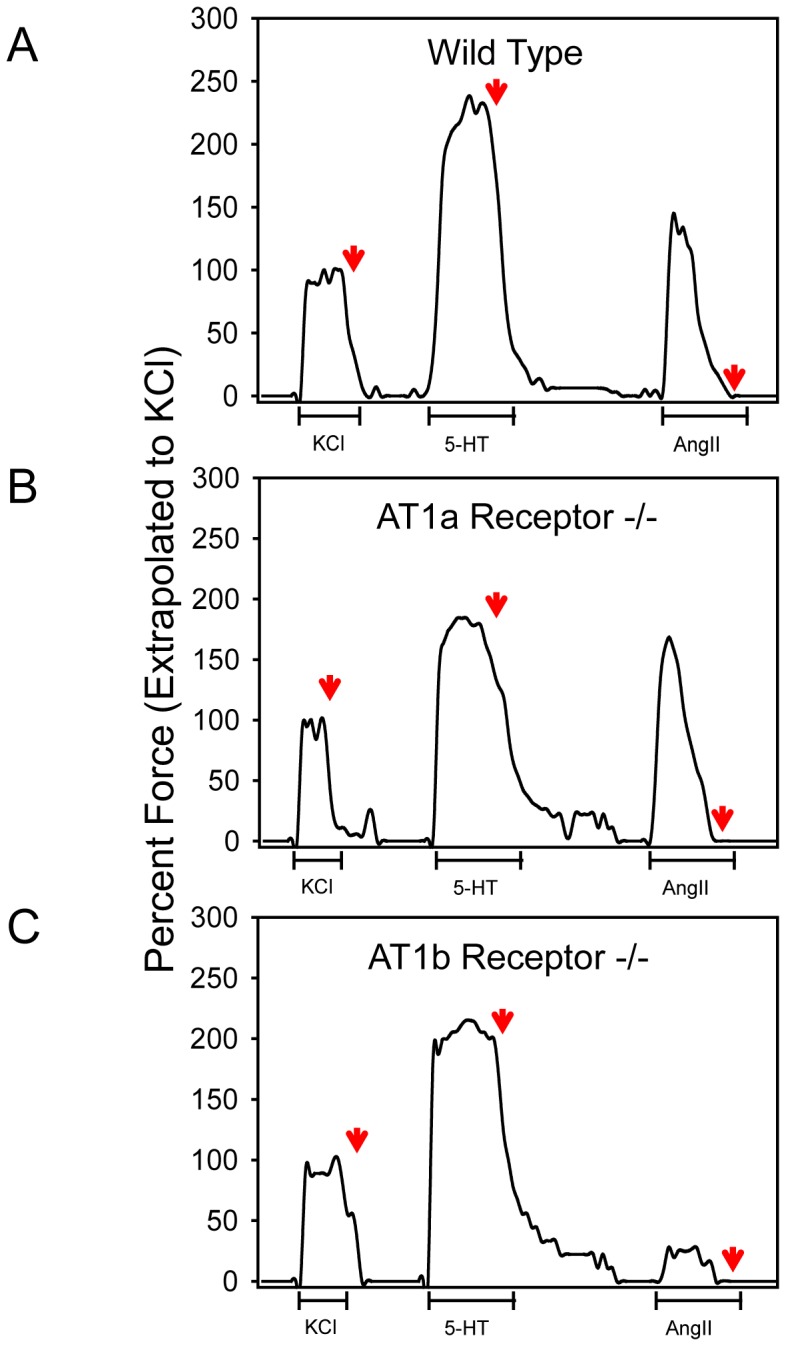
AngII-induced regional contractions were markedly reduced by AT1b receptor deficiency. Regional contractility of aortic rings harvested from the infra-renal aorta of (**A**) C57BL/6, (B) AT1a receptor −/−, or (C) AT1b receptor −/− mice. Aortic rings were contracted during 5-minute incubation with potassium chloride (KCl; 80 mM), 5-hydroxytryptamine (5-HT; 1 µM), or Ang II (1 µM). Contractions are represented as percent of the maximal contraction achieved during incubation with KCl (80 mM). Red arrows indicate the return to normal Krebs–Henseleit solution.

Given differences in regional mRNA abundance of both receptor subtypes along the length of aortic tissue, we sought to compare the role of these receptor subtypes in AngII-induced contraction. Previous studies have noted a distinction in AngII-induced contractility in thoracic and abdominal regions [3], but it has not been defined whether there were further distinctions between the receptor subtypes in abdominal aortas. After confirmation of deletion of AT1a receptor or AT1b receptor alleles by PCR, we performed contractile studies using aortic rings from aortas of C57BL/6, AT1a receptor −/−, and AT1b receptor −/− mice, respectively. As expected, KCl and 5-HT contracted infra-renal aortic rings in all genotypes (Figure 2). As reported previously, AngII only contracted rings isolated from the infra-renal region (Figure 2A) [3]. Furthermore, we confirmed that deletion of AT1a receptors had no effect on AngII-induced contractions (Figure 2B) [3]. In contrast, deficiency of AT1b receptors markedly reduced AngII-induced contraction in the infra-renal aorta (Figure 2C).

### AT1b Receptor Deficiency Had No Effect on AngII-induced Increases of SBP

A previous study inferred a role of AT1b receptors in blood pressure regulation [17] and plasma renin concentrations [29]. Following the demonstration of a functional role of AT1b receptors in aortic tissues, we infused AngII for 28 days into AT1b receptor +/+ and −/− mice. Whole body deficiency of AT1b receptors during AngII infusion had no significant effect on body weight and did not change plasma renin concentrations (Table 1). Deletion of AT1b receptors had no significant effect on SBP in response to AngII (Table 1).

**Table 1:** Characterization of AT1b receptor +/+ and −/− mice infused with AngII.

AT1b receptor genotype	+/+	−/−
	N = 16	N = 10
Body weight (g)	24.9±0.5	23.7±0.4
SBP (mmHg)	158±8	155±8
Plasma renin concentration(ng/ml/30 min)	1.87±0.6	1.56±0.6
Plasma cholesterol concentration(mg/dl)	1,690±75	1,776±64

Body weight, plasma cholesterol and renin concentrations were measured after termination. SBP was measured during the last week of the study. Values are represented as mean ± SEM. There were no significant differences between the two AT1b receptor genotypes.

### AT1b Receptor Deficiency Had No Effect on AngII-induced Aortic Atherosclerosis

**Figure 3:**
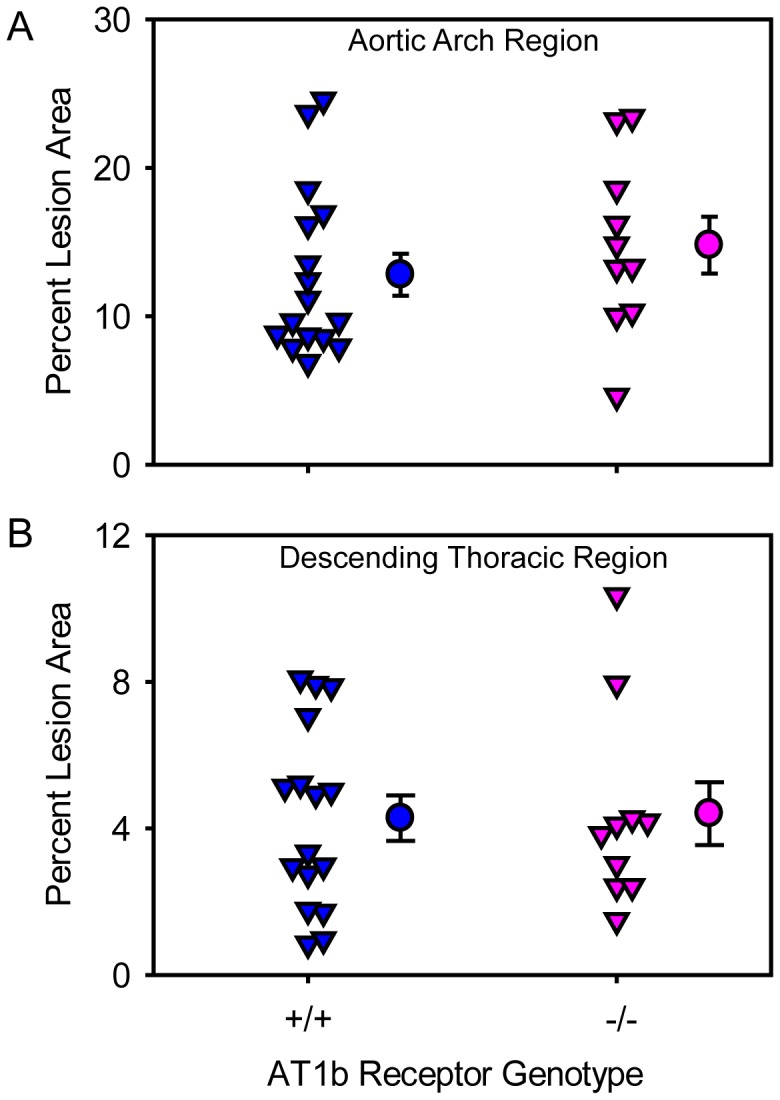
AT1b receptor deficiency had no effect on AngII-induced atherosclerosis. (**A**) Percent of atherosclerotic lesion area was measured on the intimal surface of the aortic arch region (N = 16: AT1b receptor +/+, and N = 10: AT1b receptor −/− mice). (**B**) Percent of atherosclerotic lesion area was measured on the intimal surface of the descending thoracic aorta (N = 16: AT1b receptor +/+, and N = 10: AT1b receptor −/− mice). Inverted triangles represent values from individual mice, circles represent means, and error bars are SEMs.

In male LDL receptor −/− mice that were either AT1b receptor +/+ or −/− were infused with AngII for 28 days. There was no significant difference in plasma cholesterol concentrations between the groups (Table 1). Atherosclerotic lesion size was quantified on the intimal surfaces of the aortic arch and descending thoracic regions of all mice. Abdominal aortic region was excluded because the presence of AAAs impedes accurate measurement of atherosclerosis. There was no significant difference in percent atherosclerotic lesion area in the aortic arch (Figure 3A) and descending thoracic regions (Figure 3B) of AT1b receptor +/+ and −/− mice infused with AngII. Examples of en face aortas with versus without Oil Red O staining are shown in Figure S1A and S1B, respectively.

### AT1b Receptor Deficiency Had No Effect on AngII-induced AAAs

**Figure 4:**
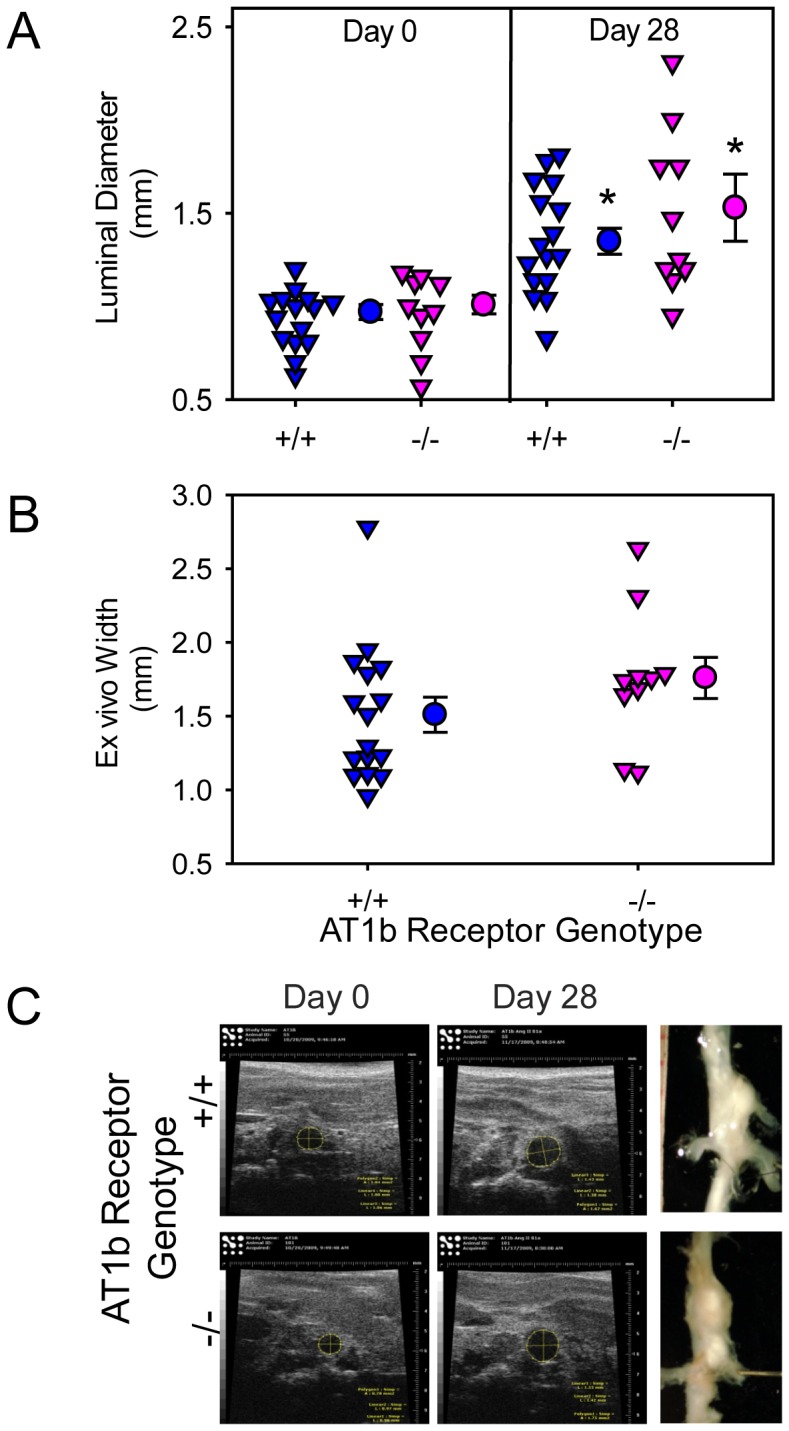
AT1b receptor deficiency had no effect on AngII-induced abdominal aortic dilation in vivo. (**A**) Maximal luminal diameters of suprarenal aortas were measured in vivo by ultrasonography at baseline (Day 0) and on Day 28 during AngII infusion (N = 16: AT1b receptor +/+, and N = 10: AT1b receptor −/− mice). * denotes P<0.001 saline versus AngII within AT1b genotypes (two way repeated measures ANOVA). (**B**) Maximum width of supra-renal aortas was measured ex vivo (N = 16: AT1b receptor +/+, and N = 10: AT1b receptor −/− mice). Inverted triangles represent individual mice, circles represent means and error bars are SEM. (**C**) Examples of ultrasound images (Day 0 and Day 28) and ex vivo pictures (after termination) of suprarenal aortas, which represent aortic diameters nearest the mean of each group.

Following the demonstration of a predominance of AT1b receptor mRNA in abdominal aortas, we sought to examine the role of AT1b receptors in AngII-induced AAA formation. Maximal luminal diameters of supra-renal aortas were determined in vivo using a high frequency ultrasound at baseline (day 0) and on day 28 during AngII infusion in LDL receptor −/− mice that were either AT1b receptor +/+ or −/−. Maximal luminal diameters significantly increased in both study groups on day 28 during AngII infusion compared to day 0 (Figure 4A; P<0.05). However, there was no significant difference in luminal diameters of supra-renal aortas between AT1b receptor +/+ and −/− mice after 28-day infusion of AngII (Figure 4A).

The presence of AAAs were confirmed by measuring maximal external width of the supra-renal region using an ex vivo method. There was no significant difference of maximal external width of supra-renal aortas between AT1b receptor +/+ and −/− mice (Figure 4B). Examples of ultrasound photographs and ex vivo images of abdominal aortas are shown in Figure 4C.

We also examined histological features of the supra-renal aorta with Movat’s pentachrome staining. There were no discernible differences of elastin fibers in supra-renal aortas prior to AngII infusion (Figure S2A). After AngII infusion, occurrence of focal elastin disruption in this aortic region was comparable between AT1b receptor +/+ and −/− mice (Figure S2B).

### AT1b Receptor Deficiency Had No Effect on AngII-induced Ascending Aortic Dilation

**Figure 5:**
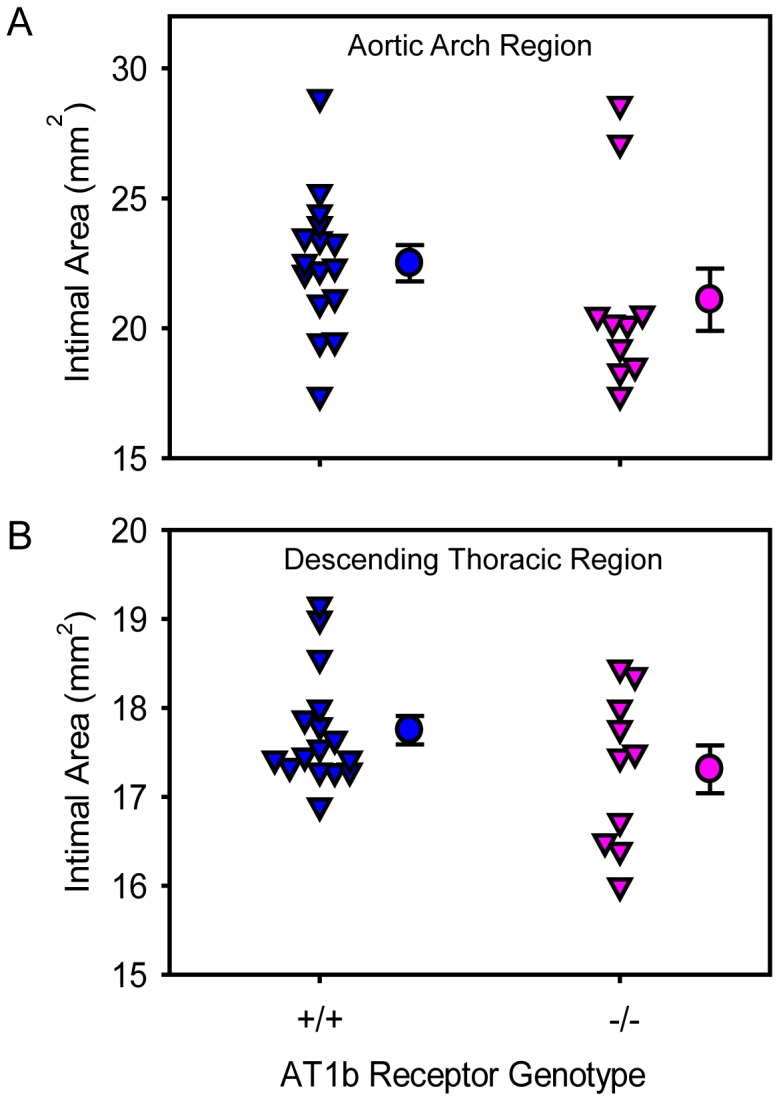
AT1b receptor deficiency had no effect on AngII-induced aortic arch and descending aortic dilation. Intimal area of (**A**) aortic arch region and (**B**) descending thoracic aorta was measured using an en face method (N = 16: AT1b receptor +/+, and N = 10: AT1b receptor −/− mice). Inverted triangles represent values from individual mice, circles represent means, and error bars are SEM.

We have recently reported that chronic AngII infusion also induces thoracic aortic aneurysms [3], [14]. In mice infused with AngII for 28 days, there was no significant difference in intimal areas of aortic arches (Figure 5A) and descending thoracic aortas (Figure 5B) between AT1b receptor +/+ and −/− mice.

## Discussion

The present study examined the role of AT1b receptors in aortic tissues in response to AngII. We initially demonstrated the concordance of the regional abundance of AT1b receptor mRNA and the presence of functional AT1b receptors in the aorta as the primary mediator of AngII-induced region-specific contractile response. Despite the presence of functional AT1b receptors in the aortic tissue, whole body deficiency of AT1b receptors had no effect on AngII-induced aortic atherosclerosis and aortic aneurysm formation in hypercholesterolemic mice.

While both AT1 receptor subtypes are present in aortic tissues, the abundance of receptor subtype mRNA has only been characterized in selected aortic regions [30], [31]. In the present study, we demonstrated that both receptor subtypes exhibit regional differences in mRNA abundances in the aorta. We confirmed that mRNA of AT1b receptors was significantly more abundant in the abdominal region compared to the thoracic region [2], [31], and extended previous findings by demonstrating that the AT1b receptor subtype in the infra-renal region had much greater abundance than in the supra-renal area. AT1a receptor mRNA was detected in liver and kidney; however, AT1b receptor mRNA was absent or negligible in these tissues. Our results are in agreement with previous studies that AT1a receptors are more ubiquitously expressed and AT1b receptors are restricted to some tissues [2], [5], [8].

AngII is known to promote many physiological and pathological responses in a region-specific manner in the aorta [2], [31]–[33]. This may be due to heterogeneous developmental origins of SMCs [34]. Furthermore, it has been demonstrated that contractile responses to AngII differ in selected regions of the aorta [2], [3], [8], [30], [35]. In the present study, we sought to examine the contractile responses to AngII in infra-renal aortic tissues in the presence or absence of AT1a and AT1b receptors. We have noted previously that there are minimal contractile responses to AngII in ascending and descending thoracic aortas [3]. AngII promoted contraction of the infra-renal aorta in C57BL/6 mice. Despite higher abundance of AT1a receptors in the infra-renal portion of the abdominal aorta, deletion of AT1a receptors had no effect on contractile responses to AngII. In contrast, the contractile response to AngII was diminished in infra-renal aortas from AT1b receptor −/− mice. This effect coincided with the greatest abundance of AT1b receptor mRNA. Our results are in agreement with a previous study that indirectly demonstrated AngII-induced contractile responses being linked to AT1b receptors through the use of an AT1 receptor antagonist (losartan) in AT1a receptor −/− mice [2]. In another study, AngII-induced contractile responses were defined in AT1b receptor +/+ and −/− mice and demonstrated that AT1b receptors mediated contraction of abdominal aortas [11]. The present study demonstrated that the regional specificity of AngII-induced aortic contractions coincided with the greatest abundance of AT1b receptor mRNA. However, there are still quandaries relating to these data. AT1a receptor mRNA was also most abundant in this region, but this receptor subtype did not affect AngII-induced contractions in this region. Additionally, expression of both receptors was detected in the supra-renal aorta, but this region does not contract in response to AngII stimulation [3].

Blood pressure is considered an important risk factor for cardiovascular diseases. A previous study that examined residual pressor effects of AngII in AT1a receptor −/− mice suggested that AT1b receptors might contribute to pressor effects in the absence of AT1a receptors [17]. However, it has been demonstrated previously that whole body deficiency of AT1a receptors ablates AngII-induced increases of systolic blood pressure [15], [29]. The location of AT1a receptors that mediates the increased blood pressure during AngII infusion has not been defined. Recent reports provided surprising results that depletion of AT1a receptors using SM22-promoter driven Cre did not affect AngII-induced increases in systolic blood pressure [3]. Despite not being clearly defined, this apparent paradox may be related to the lack of expression of SM22-driven Cre in the kidney that is a major tissue controlling AngII-induced hypertension [36], [37]. In the present study, deletion of AT1b receptors had no effect on blood pressure induced by infusion with AngII. Others have also demonstrated that AT1b receptor deletion had no effect on AngII-induced increases in SBP [18]. Overall, despite the requirement of AT1b receptors for contractile responses to AngII in the infra-renal aorta, we were unable to demonstrate an effect of AT1b receptors on blood pressure regulation. As with AT1a receptors, the contrasting effect of AT1b receptor deficiency on AngII-induced aortic contraction versus systolic blood pressure presumably reflects the vascular bed specific effects of AngII.

Hypercholesterolemic mice infused with AngII for 4 weeks develop aortic atherosclerosis predominantly in the ascending aortic region [14], [15], [38], [39], while the development of lesions in the abdominal aortic region is modest unless AngII infusion is prolonged [13]. Aortas display a gradient of mRNA abundance for both AT1a and AT1b receptors that was lowest in the ascending region and highest in the infra-renal region. Although the ascending aortic region has the lowest abundance for both AT1a and AT1b receptor mRNA, this is the major site for initiation and propagation of atherosclerosis [40], [41]. Our results showed that absence of AT1b receptors had no effect on the size of AngII-induced atherosclerotic lesions in either the aortic arch or the descending thoracic region in LDL receptor −/− mice, indicating that AT1b receptors do not influence the development of atherosclerosis. However, despite the low abundance of AT1a receptor mRNA in the ascending aorta, deficiency of this receptor ablated AngII-induced atherosclerosis in this region [15]. Therefore, development of atherosclerosis was not associated with the regional abundance of mRNA of either AngII type 1 receptor subtype.

To directly determine the role of AngII stimulation of AT1b receptors in pathological processes of aortic aneurysm formation, we infused AngII into AT1b receptor +/+ and −/− mice for 28 days. Despite the presence of AT1b receptor mRNA in this region, deletion of this gene had no effect on AngII-induced AAA formation. In agreement with previous findings, these results demonstrate that AT1a receptors are responsible for AngII-induced AAA formation [15]. AngII infusion promotes pronounced ascending aortic dilation [14] and mRNA of both AT1 receptor subtypes are expressed in this region. However, deletion of AT1b receptors had no effect on, while whole body deficiency of AT1a receptors ablated, ascending aortic aneurysms [3]. Therefore, the relative regional mRNA abundance of both AT1 receptor subtypes has no obvious relationship to the location of AngII-induced aortic pathologies.

In summary, there are several major findings in the present study including AT1b receptors contributing to AngII-induced aortic contraction that parallels with the mRNA abundance of AT1b receptors in this region, and AT1b receptors not contributing to AngII-induced atherosclerosis and aortic aneurysms in either the ascending or abdominal aortic region. Interestingly, AT1a and AT1b receptors are abundant in the infra-renal region but exhibit different vascular contractility with AngII stimulation. These pathophysiological differences may be due to amino acid differences in the C-terminal region between the two receptor subtypes, resulting in elicitation of distinct signaling pathways. Future studies will determine whether relative variations in the protein sequences of these two receptor subtypes lead to differential responses to AngII in order to understand the different modes of action induced by the two receptor subtypes in AngII-induced vascular diseases.

## Supporting Information

Figure S1 **Images of en face aortas.** (**A**) Examples of en face aortas without any staining. Atherosclerotic lesions of which represent values near the mean of each group. (**B**) En face aortas with Oil Red O staining. These aortas are the same as shown in (**A**).
(PDF)

Figure S2 **Movat’s pentachrome staining of supra-renal aortic sections.** (**A**) Images of supra-renal aortas from AT1b receptor +/+ or −/− mice prior to AngII infusion (40X). (**B**) Images of AAAs from AT1b receptor +/+ or −/− mice after 28 days of AngII infusion (40X).
(PDF)
