# Supplementary material for: Regional Variation in Aortic AT1b Receptor mRNA Abundance Is Associated with Contractility but Unrelated to Atherosclerosis and Aortic Aneurysms
Source: PLoS One. 2012 Oct 31;7(10):e48462. doi: 10.1371/journal.pone.0048462 (PMC3485205; doi:10.1371/journal.pone.0048462)
Supplement: Figure S1 — Images of en face aortas. (A) Examples of en face aortas without any staining. Atherosclerotic lesions of which represent values near the mean of each group. (B) En face aortas with Oil Red O staining. These aortas are the same as shown in (A). (PDF) [file pone.0048462.s001.pdf]

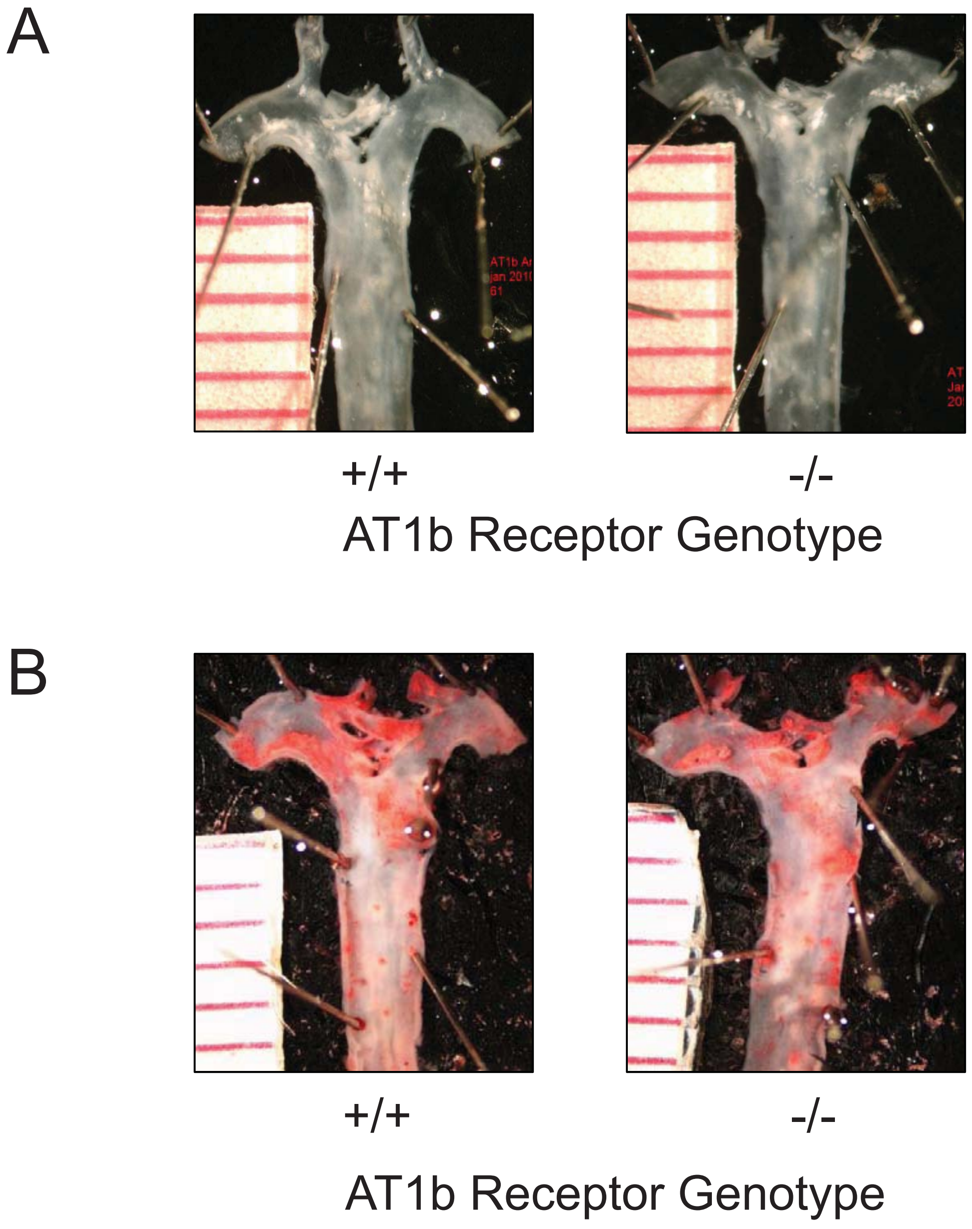

Figure S1. Images of en face aortas. (**A**) Examples of en face aortas without any staining. Atherosclerotic lesions of which represent values near the mean of each group. (**B**) En face aortas with Oil Red O staining. These aortas are the same as shown in (**A**).
