# Supplementary material for: Regional Variation in Aortic AT1b Receptor mRNA Abundance Is Associated with Contractility but Unrelated to Atherosclerosis and Aortic Aneurysms
Source: PLoS One. 2012 Oct 31;7(10):e48462. doi: 10.1371/journal.pone.0048462 (PMC3485205; doi:10.1371/journal.pone.0048462)
Supplement: Figure S2 — Movat’s pentachrome staining of supra-renal aortic sections. (A) Images of supra-renal aortas from AT1b receptor +/+ or −/− mice prior to AngII infusion (40X). (B) Images of AAAs from AT1b receptor +/+ or −/− mice after 28 days of AngII infusion (40X). (PDF) [file pone.0048462.s002.pdf]

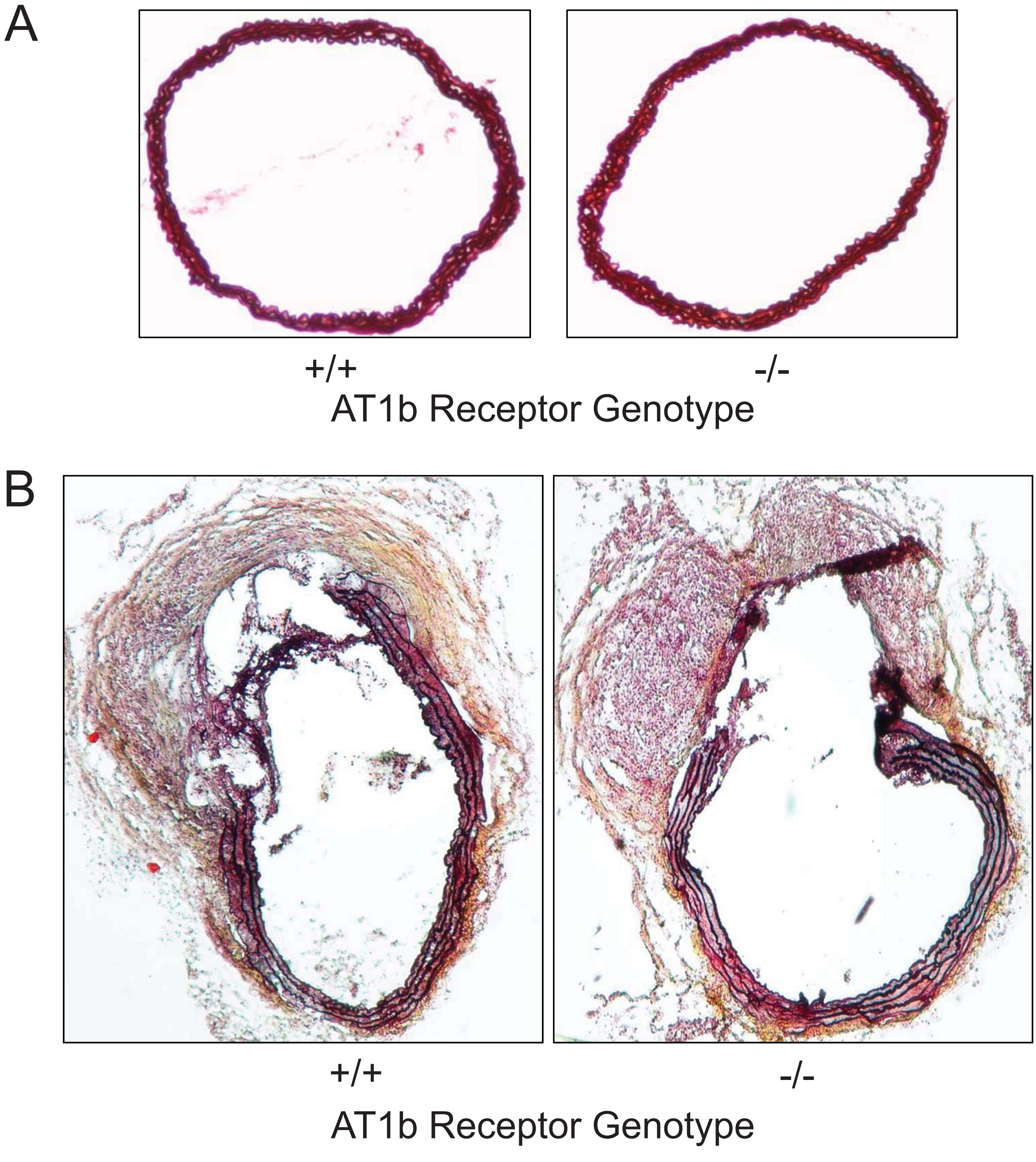

Figure S2. Movat's pentachrome staining of supra-renal aortic sections. (**A**) Images of supra-renal aortas from AT1b receptor +/+ or -/- mice prior to AngII infusion (40X). (**B**) Images of AAAs from AT1b receptor +/+ or -/- mice after 28 days of AngII infusion (40X).
